# Long-Acting Real-Time Microscopic Monitoring Inside the Proton Exchange Membrane Water Electrolyzer

**DOI:** 10.3390/s23125595

**Published:** 2023-06-15

**Authors:** Chi-Yuan Lee, Chia-Hung Chen, Hsian-Chun Chuang, Hsiao-Te Hsieh, Yen-Chen Chiu

**Affiliations:** 1 Department of Mechanical Engineering, Yuan Ze Fuel Cell Center, Yuan Ze University, Taoyuan 32003, Taiwan; 2 Homytech Global Co., Ltd., Taoyuan 33464, Taiwan

**Keywords:** PEMWE, MEMS, flexible 7-in-1 microsensor, 200 h accelerated aging test, temperature difference, current drop

## Abstract

The proton exchange membrane water electrolyzer (PEMWE) requires a high operating voltage for hydrogen production to accelerate the decomposition of hydrogen molecules so that the PEMWE ages or fails. According to the prior findings of this R&D team, temperature and voltage can influence the performance or aging of PEMWE. As the PEMWE ages inside, the nonuniform flow distribution results in large temperature differences, current density drops, and runner plate corrosion. The mechanical stress and thermal stress resulting from pressure distribution nonuniformity will induce the local aging or failure of PEMWE. The authors of this study used gold etchant for etching, and acetone was used for the lift-off part. The wet etching method has the risk of over-etching, and the cost of the etching solution is also higher than that of acetone. Therefore, the authors of this experiment adopted a lift-off process. Using the flexible seven-in-one (voltage, current, temperature, humidity, flow, pressure, oxygen) microsensor developed by our team, after optimized design, fabrication, and reliability testing, it was embedded in PEMWE for 200 h. The results of our accelerated aging test prove that these physical factors affect the aging of PEMWE.

## 1. Introduction

Variable Renewable Energy (VRE) has seen tremendous growth over the past decade, with solar up at least 1800% and wind around 300% [1]. The complementary capabilities of PEMWE and VRE make it an important asset for the production of green energy in industrial applications. The main water electrolysis technologies include Alkaline Water Electrolysis (AWE), Proton Exchange Membrane Water Electrolysis (PEMWE), and Solid Oxide Electrolysis (SOEC) [2]. Hydrogen energy is a widely accepted alternative energy source with great potential to reduce our dependence on petroleum, air pollution, and greenhouse gas emissions, making it a viable way to create clean energy [3]. The production, storage, and utilization of hydrogen cycles have been studied extensively. Among various kinds of manufacturing technologies (e.g., bioproduction, steam reforming), the water electrolyzer is essential in the research and development of energy-related techniques due to its application in renewable energy [4]. The capability of a hydrogen energy system in power grid and microgrid scale grows increasingly. The concept of hydrogen economy has been in development for a few decades, but the hydrogen value chain has not demonstrated its feasibility in commercial applications until recently. The most important factors that push this trend include the significantly reduced cost of solar and wind technologies and the continuous improvement of hydrogen energy technology and supporting infrastructure. In addition, many advanced countries have stressed the importance of developing hydrogen energy to the world energy requirement and supply chain. 

In Europe, traffic accounts for about 1/3 of total energy consumption and about 1/4 of total emission of greenhouse gases, most of which comes from vehicles on roads [5]. Over the past decade, more people have paid considerable attention to the development of electric vehicles (EVs). In order to reduce greenhouse gas emissions, battery electric vehicles (BEVs) and fuel-cell electric vehicles (FCVs) have been widely researched and developed. Due to the zero-carbon-emission-emitting capacity of these vehicles, the electric power generated from renewable energy and oxygen utilization are expected to reduce the worldwide total of carbon emissions [6,7]. BEV is a mature technology used in many advanced countries at present, while FCV is considered to be a part of future technology. In recent years, the quantity of BEVs has increased worldwide, especially in China, the U.S., and Europe, but the technology has a very serious bottleneck. The main difficulties include (i) short driving distance, (ii) long charging time, (iii) high cost of investment, and (iv) finite infrastructure. The battery is the foremost challenge with BEVs. In order to increase the running kilometrage of a BEV, the battery capacity must be increased; however, this also increases the vehicle’s weight, thereby reducing its efficiency. The main advantage of hydrogen is its high energy density. Thus, the development of hydrogen FCVs is highly expected. Fuel cells have been produced on a large scale in recent years. From portable electronic products, mobile carriers, and forklifts to stationary power generation, the hydrogen fuel cell has been commercialized in different areas [8]. The demand for hydrogen has increased by over three times since 1975, and many countries are actively studying the viability of using hydrogen energy in industry or to improve the lives of the general public. It is anticipated that hydrogen energy will be the leading form of energy in the energy market in the near future and that the hydrogen economy will become a trend. 

The hydrogen can be generated by a solid oxide electrolyzer (SOE), but the SOE is deficient in terms of stability and likely to degrade, and it works at temperatures as high as 500 °C. The high temperature consumes more energy and generates excess waste heat, which is likely to harm the environment if not properly disposed of [9]. The proton exchange membrane water electrolyzer (PEMWE) is the most advanced water electrolyzer type and it is a more promising electrochemical appliance with maximum efficiency, environmental protection, and high cost benefits for hydrogen production compared to conventional electrolyzers and hydrocarbon steam reformers [10]. Early in the 1950s, Grubb proposed the PEMWE for the first time (which was further developed by GEC in 1966) and overcame the problems associated with alkaline electrolyzers [11]. The PEMWE can generate very pure hydrogen under high pressure [12]. In addition, the PEMWE is characterized by working at a high current density (>2 A cm^−2^) and low temperature (20~80 °C), generating a low carbon footprint and quick response. 

The PEMWE has many advantages over conventional electrolyzed water hydrogen production techniques, such as higher yield, simpler system, higher energy efficiency, and lower operating temperature [13]. The PEMWE provides additional electric energy, which is converted into hydrogen energy via an electrochemical reaction, and the water is directly decomposed into hydrogen and oxygen. The anode terminal equation is:

2H_2_O → O_2_ + 4H^+^ + 4e (1)

The electrons of the anode reaction reach the cathode via the external circuit, and the hydrogen ions reach the cathode via the proton exchange membrane. The cathode equation is expressed as:

2H^+^ + 2e^−^ → H_2_ (2)

Equation (1) is combined with Equation (2) to obtain the net reaction of PEMWE:

2H_2_O → 2H_2_ + O_2_ (3)

There are seven important physical quantities associated with the operation of the PEMWE: oxygen, voltage, current, temperature, humidity, flow, and pressure, all of which have a critical influence on its aging and lifespan. If the flow of a PEMWE is too low, the hydrogen production efficiency will be reduced. This is because a non-uniform flow distribution can damage the anticorrosion microporous layer, which leads to an increase in overall impedance, a decrease in performance, and accelerated aging. If the temperature of the membrane electrode assembly (MEA) is not able to reach the optimal working temperature, the performance of the PEMWE cannot be improved. Additionally, the MEA may be damaged if the temperature is too high, and the bipolar plate material may become corroded and dissolved in a harsh high-voltage chemical environment. Sartory et al. [14] found that the galvanic pile efficiency and total efficiency of the PEMWE decreased with pressure, and the temperature could reduce the efficiency. Lu et al. [15] studied the PEMWE polarization curve and found that the voltage rose very fast in the case of low current as the current density increased and then slowed down before rising nonlinearly. Frensch et al. [16] reported that the PEMWE worked efficiently at 90 °C, but the fluoride emission rate increased, and the membrane thinned with time. The thinner membrane increased gas cross, but the higher temperature shortened the membrane life. Aßmannet et al. [17] found that the PEMWE used an Ir-Ru oxide catalyst as an anode and that the decay rate tripled after 1000 h of operation at fixed 3 A cm^−2^. Onana et al. [18] studied the PEMWE aging degrees at 60 °C and 80 °C and found that the oxide grew fast at high temperatures. The overall battery resistance increased, and the membrane thinned while the aging was accelerated. Siracusano et al. [19] found that the MEA manufacturing process and method could influence the performance and lifespan of the PEMWE. Müller et al. [20] used MEAs in different thicknesses to compare the performance of the PEMWE. The experimental results showed that the thinner membrane material had better performance in terms of efficiency and power. Verdin et al. [21] indicated that the PEMWE pressure distribution uniformity could influence the current distribution uniformity and that any mechanical stress and thermal stress induced by nonuniformity might accelerate local aging of the PEMWE. It is known from the existing literature that the amount of oxygen produced by the PEMWE is determined by the current density, and under the condition of accelerated aging at constant voltage, the control of temperature, pressure, and water flow will all affect the current density and then affect the oxygen production efficiency and humidity distribution of PEMWE. Therefore, a flexible seven-in-one (voltage, current, temperature, humidity, flow, pressure, oxygen) microsensor was developed and embedded in the PEMWE to measure accelerated aging data.

## 2. Materials and Methods

### 2.1. Development of Flexible Micro-Oxygen Sensor

**Figure 1:**
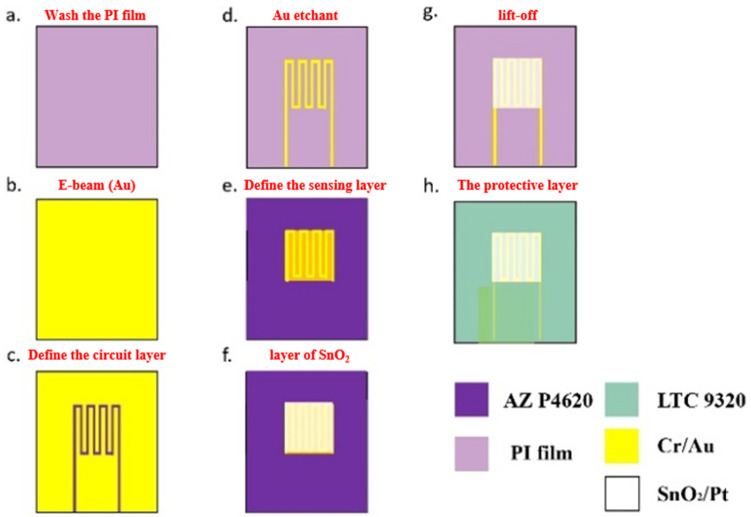
Manufacturing process chart of flexible micro-oxygen sensor.

We used MEMS technology to develop a semiconductor type micro-oxygen sensor, mostly matching the process integration technology of the research team behind ref. [20]. Because etching is better, it was used. The manufacturing process is shown in Figure 1 and was as follows:

(a) Wash the PI film with acetone and methanol organic solutions, respectively. Use DI water to remove residual methanol, repel surface dust and residual grease, and increase the adhesion of thin film metal.
(b) Then, use an E-beam evaporator to first vapor-deposit Cr as an adhesive layer between Au and PI film to promote the adhesion between gold and PI film, and then use a plating rate of 0.1 Å/s to Complete the deposition step of gold with a thickness of 1200 Å.
(c) Spin-coat AZ P4620 (positive photoresist), expose and develop to define the electrode pattern of the micro-oxygen sensor.
(d) Use Au etchant (Type-TFA) to etch Au, then use Cr etchant (Cr-7T) to etch Cr, and remove the photoresist that was originally used as an etching mask with Remove 1165.
(e) Then, spin coat AZ P4620 once again as a spray mask. The micro-oxygen sensor pattern is defined by exposure; the required pattern can be developed by developer.
(f) The sputtering machine is used to deposit a thin layer of SnO_2_ onto the surface of the pattern, which will serve as the gas sensing layer, and a layer of Pt is then deposited onto the SnO_2_ layer, which will serve as the catalyst layer.
(g) The sample should then be immersed in 80 °C photoresist remover (Remove 1165) for 20 min. Afterwards, the photoresist mask should be removed using lift-off, followed by cleaning with acetone and methanol.

To complete the fabrication, spin coat the LTC 9320 (negative photoresist) on the flexible micro-oxygen sensor. 

### 2.2. Process Integration Development of Flexible Seven-in-One Microsensor

**Figure 2:**
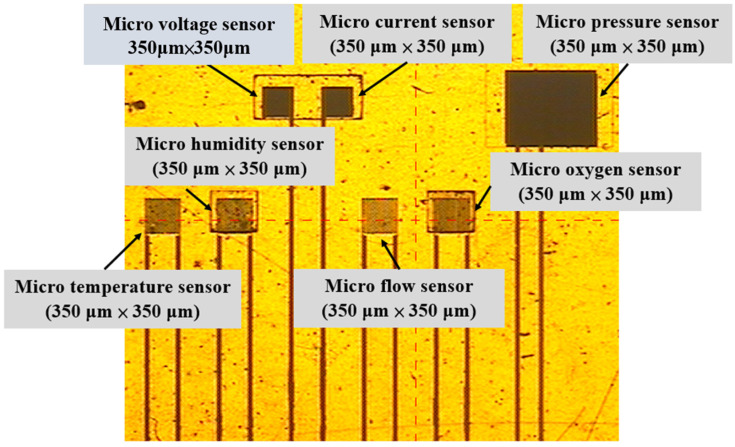
Flexible 7-in-1 microsensor lithography.

Using MEMS technology, a flexible micro-oxygen sensor was successfully developed, and the flexible micro-oxygen sensor was integrated with other microsensors to manufacture a flexible 7-in-1 microsensor. This sensor is capable of measuring multiple parameters simultaneously, making it a seven-in-one microsensor. The manufacturing process of the flexible seven-in-one microsensor roughly included the following steps, and the completed diagram is shown in Figure 2.

First, the PI film was cleaned with organic solutions of methanol and acetone. After that, the residual methanol was removed by DI water, followed by the removal of surface dust, residual oil, and fat in order to increase the adhesion of the metal thin film. The Au and Cr layers were evaporated on the substrate using an E-beam evaporator for use as an adhesion layer and sensing electrode layer. The patterns of micro-oxygen, voltage, current, temperature, humidity, pressure, and flow sensors were defined by using photolithography. Cr etching solution and Au etching solution were the chemical solutions used for wet etching. The photoresist was coated, and the micro-oxygen sensor pattern was defined. The photoresist mask was removed, and the SnO_2_ and Pt were sputtered on the micro-oxygen sensor. The LTC 9320 was used as a protective coating, and the sensing areas and pins of micro voltage, current, humidity, and pressure sensors were defined through the use of photolithography, leaving them exposed and not covered by the protective layer. The LTC 9305 is a thin film coating used to construct a micro humidity sensor. This coating was then used to construct a flexible seven-in-one microsensor, as shown in Figure 2. This microsensor is highly sensitive to humidity.

## 3. Results and Discussion

### 3.1. PEMWE Real-Time Microscopic Monitoring

**Figure 3:**
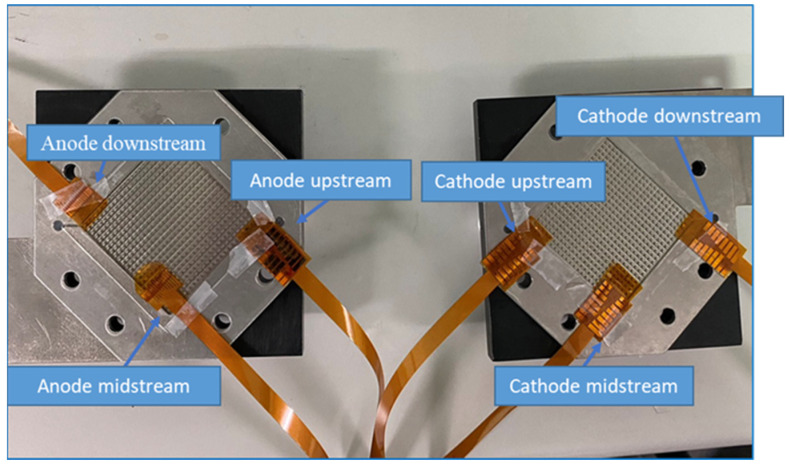
Flexible seven-in-one microsensor embedded in the position of anode and cathode.

We used Homytech to develop a PEMWE, including the development of the PEMWE’s structure, flow field design, and selection of material. The flow field design used a columnar runner, which can carry gases away rapidly. Because oxygen can be quickly removed, the presence of gas in the flow channel did not decrease the area available for the water electrolysis reaction. The assembly of a flexible seven-in-one microsensor and the PEMWE was completed with the collector plate designed in this study and by referring to the assembly processes of related techniques. The stereogram is shown in Figure 3. 

#### 3.1.1. PEMWE Real-Time Microscopic Monitoring

A 200 h test was conducted to observe the differences in the internal state of Proton Exchange Membrane Water Electrolysis (PEMWE) and its associated Membrane Electrode Assembly (MEA) by embedding a flexible seven-in-one microsensor in the downstream, midstream, and upstream of the PEMWE and MEA.

#### 3.1.2. PEMWE Testing Environment

At 25 °C, a flow velocity of 90 mL/min and a constant voltage of 2 V were used to operate the PEMWE at a pressure of 3.2 bar. A flexible seven-in-one microsensor and an NI PXI cabinet with high-accuracy capture equipment were used to monitor the PEMWE internally and locally in real-time. 

#### 3.1.3. PEMWE Voltage Test

**Figure 4:**
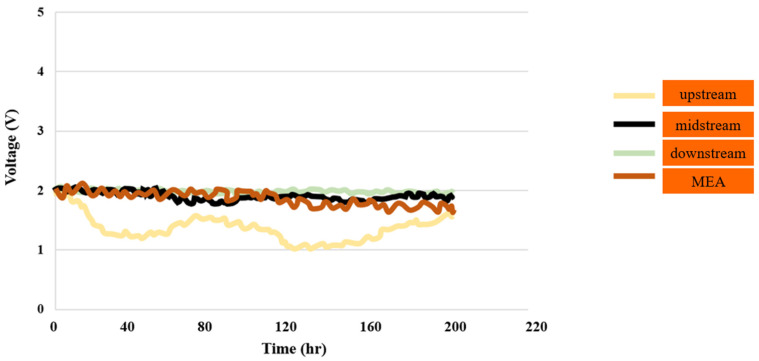
Voltage distributions in downstream, midstream, and upstream inlet, as well as in MEA, for up to 200 h.

The flow velocity was 90 mL/min, while the temperature of the DI water fed into the PEMWE was 25 °C. Measurements were taken at a constant voltage of 2 V. Figure 4 shows the voltage distributions for the upstream inlet, midstream inlet, downstream inlet, and MEA. As can be seen, there was a significant and noticeable alteration in the inlet point further upstream.

#### 3.1.4. PEMWE Current Test

**Figure 5:**
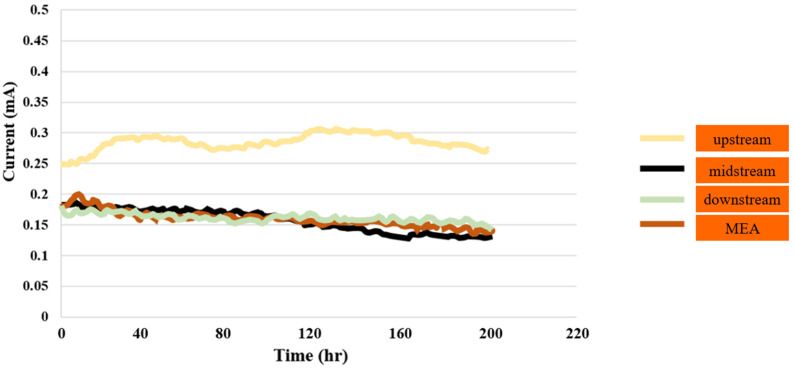
Current distributions in downstream, midstream, and upstream inlet, as well as in MEA, for up to 200 h.

The current distributions in the downstream, midstream, upstream, and MEA were measured for 200 h with a constant voltage of 2 V, and the signals were captured every 30 min at a flow velocity of 90 mL/min and an environment temperature of 25 °C. Figure 5 shows the current variation in the upstream inlet, which is relatively apparent in the reaction process.

#### 3.1.5. PEMWE Temperature Test

**Figure 6:**
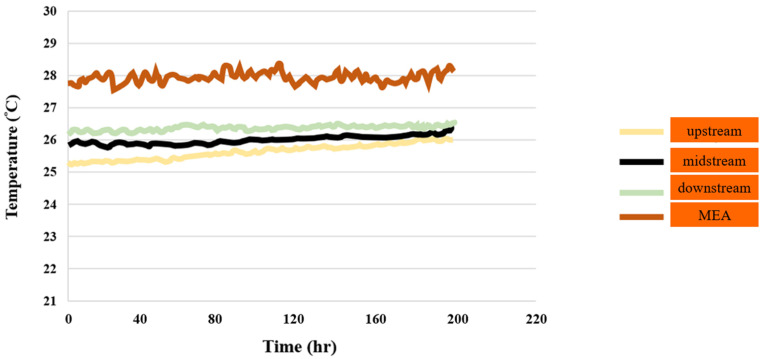
Temperature distributions in downstream, midstream, and upstream inlet, as well as in MEA, for up to 200 h.

The data indicate that there was a significant temperature variation in the upstream area during the reaction process, whereas the temperature changes in the midstream and downstream areas were comparatively minor. This was because the water in the PEMWE generated some heat during the reaction, resulting in a slight temperature increase that was conducted downstream. This is illustrated in Figure 6.

#### 3.1.6. PEMWE Humidity Test

**Figure 7:**
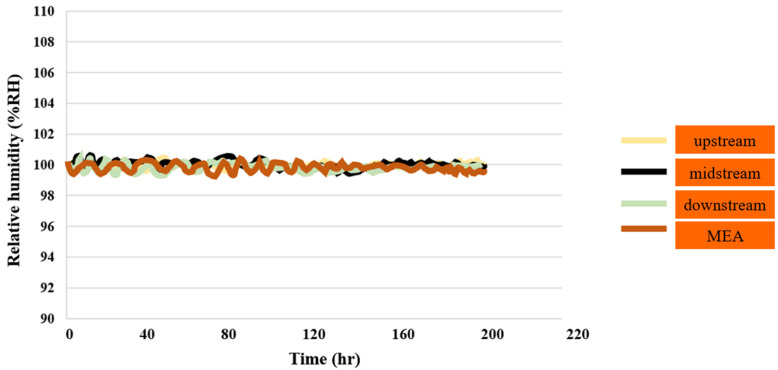
Humidity distributions in downstream, midstream, and upstream inlet, as well as in MEA, for up to 200 h.

The data indicate that the relative humidity in the upstream, midstream, downstream, and MEA was 100% RH during the reaction process. However, the resistive micro-humidity sensor had some errors due to temperature effects, as illustrated in Figure 7.

#### 3.1.7. PEMWE Flow Test

**Figure 8:**
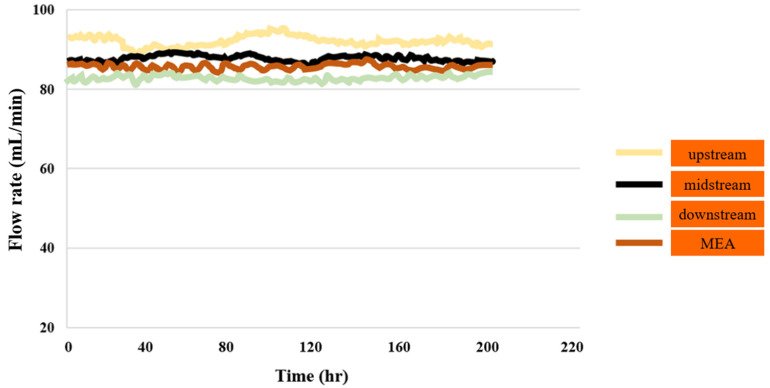
Flow distributions in downstream, midstream, and upstream inlet, as well as in MEA, for up to 200 h.

Figure 8 shows the flow distribution of PEMWE and MEA during 200 h of operation. It can be seen that the flow velocity in the upstream area of the flow channel is the highest while that in the downstream area is the lowest. This is because the columnar runner has a smoother and more stable flow compared to the snake-like runner.

#### 3.1.8. PEMWE Pressure Test

**Figure 9:**
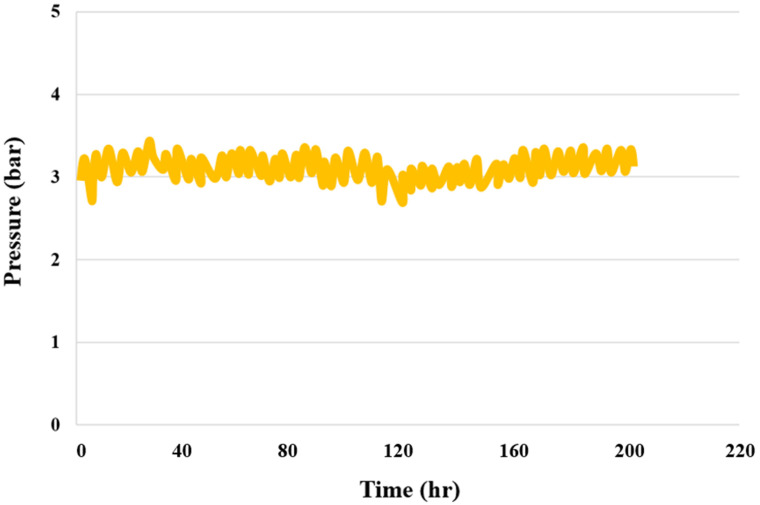
Pressure distribution in the upstream area for up to 200 h.

Figure 9 shows the results of the 200 h upstream PEMWE pressure test. The pressure was fixed at three bars for the duration of the test. There were no significant changes in the internal pressure, indicating that the design and assembly of the PEMWE are good and that there are no leaks present.

#### 3.1.9. PEMWE Oxygen Test

**Figure 10:**
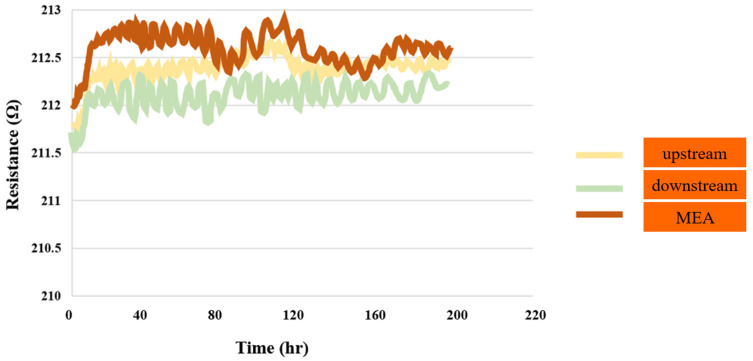
Oxygen distributions in downstream, midstream, and upstream inlet, as well as in MEA, for up to 200 h.

Figure 10 shows the results of the 200 h test for the performance of the PEMWE in the downstream and upstream areas of the anode and MEA. The micro-oxygen sensors in the downstream and upstream areas of the anode fail to detect any oxygen, while in the MEA, the partial cathode terminal hydrogen is detected. The resistivity curve of the partial hydrogen is relatively sharp in comparison to the lack of oxygen detection.

### 3.2. Accelerated Aging Performance Comparison of PEMWE

This team successfully performed the long-term monitoring of PEMWE in the past. The prior results from our research and development team showed that the information of internal and local current, voltage, humidity, temperature, pressure, flow, and hydrogen of PEMWE was captured successfully by the 100 h long-term monitoring of PEMWE under 1.8 V. The operational process of PEMWE in 1.8 V and 100 h environment was good [22]. In this study, based on past experience, a flexible seven-in-one microsensor was embedded in the MEA of PEMWE to monitor the internal state, and the voltage and current changes in PEMWE were measured under different temperature, pressure, and flow changes, and the results are described in this paper. The data were collected to form the polarization curve of PEMWE.

#### 3.2.1. Temperature Rise Test for PEMWE 

**Figure 11:**
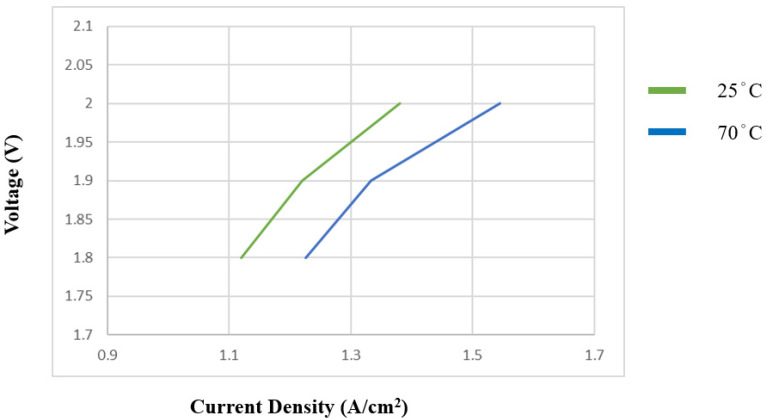
Influence of temperature on PEMWE.

Figure 11 shows the polarization curve of PEMWE at different temperatures. As can be seen, the current density increased as the PEMWE temperature rose. The results show that the rise in temperature can increase the efficiency of PEMWE.

#### 3.2.2. Pressure Variation Test for PEMWE 

**Figure 12:**
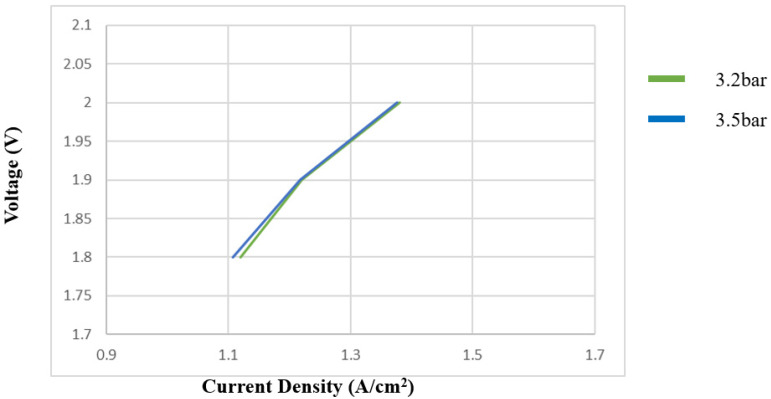
Influence of pressure on PEMWE.

Figure 12 shows the polarization curve of PEMWE under different pressures. As can be seen, the current density decreased as the PEMWE pressure increased. The boosting environment can stabilize the PEMWE in the accelerated aging environment. 

#### 3.2.3. Flowrate Variation Test for PEMWE

**Figure 13:**
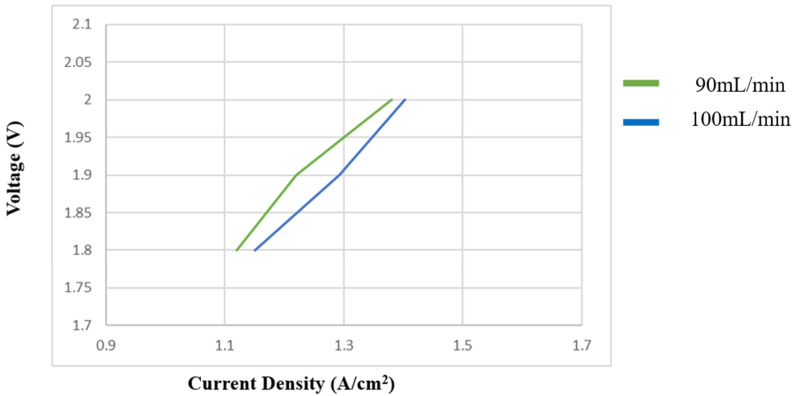
Influence of flow on PEMWE.

Figure 13 shows the polarization curve of PEMWE at different flow rates. As can be seen, the current density increased as the PEMWE flow increased. The results show that the increase in flow can increase the efficiency of PEMWE. 

#### 3.2.4. Boosting Test for High-Pressure PEMWE at Different Flow Rates 

**Figure 14:**
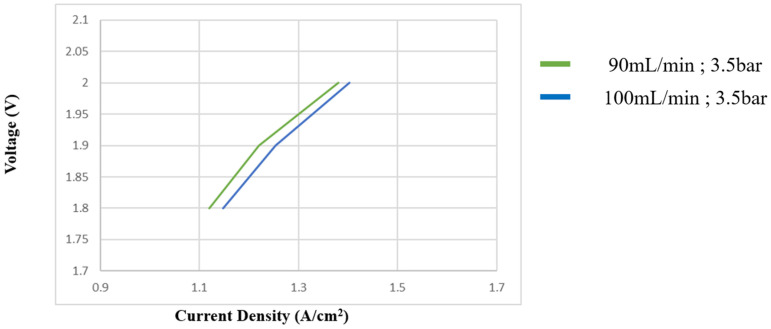
Influence of boosting at different flow rates on PEMWE.

**Figure 15:**
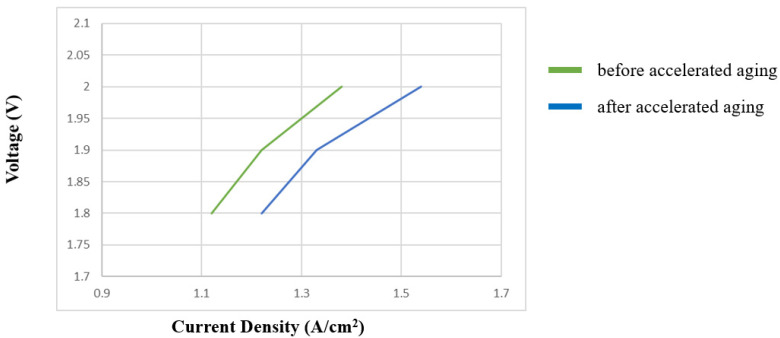
Voltage decay data before and after accelerated aging.

Figure 14 shows the influence of boosting at different flow rates on PEMWE. The PEMWE current density changed drastically when the flow increased; therefore, the pressure increased as the flow increased. In comparison to Figure 15, the current density under the 3.5 bar is steadier than that under the 3.2 bar.

#### 3.2.5. PEMWE Voltage Decay

Figure 15 shows the polarization curves before and after the accelerated aging of PEMWE. As can be seen, the voltage decay amplitude before and after accelerated aging was about 10%, but the efficiency part can be supplemented by the temperature, flow, and pressure parts.

## 4. Conclusions

This study developed a flexible microsensor by using MEMS technology and a Polyimide substrate that can measure seven different parameters: temperature, humidity, voltage, current, pressure, flow, and oxygen. 

The microsensor is small, highly sensitive, corrosion-resistant, and has good temperature tolerance due to the presence of a protective layer on the PI substrate. It can be used in various orientations and provides real-time measurements.

**Figure 16:**
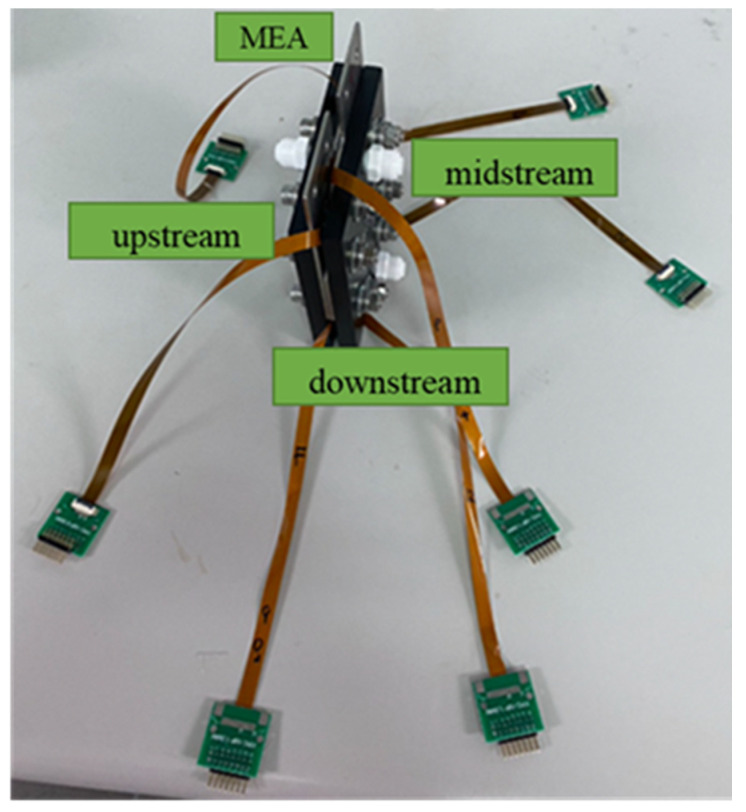
Stereogram of flexible seven-in-one microsensor embedded in PEMWE.

During an experiment involving a Proton Exchange Membrane Water Electrolyzer (PEMWE), four flexible microsensors were successfully inserted into different positions within the anode runner plate and Membrane Electrode Assembly (MEA) without affecting the PEMWE’s operation. As alluded to in Figure 16, the flexible sensor does not leak after the PEMWE lock is completed.

These sensors were able to collect important information regarding temperature, humidity, voltage, current, pressure, flow, and oxygen levels within the PEMWE over a 200 h period of accelerated aging.

The data acquired revealed that temperature, flow, and pressure had an impact on the accelerated aging of the PEMWE. Specifically, temperature had the greatest influence on the efficiency of the PEMWE in the accelerated aging environment. Additionally, boosting was found to be helpful in maintaining a consistent current density variation during the accelerated aging process. An increase in PEMWE voltage will increase the production efficiency of gas; thus, the flow rate must be increased to prevent gas from accumulating in the flow channel.

## Data Availability

Not applicable.
